# Efficacy of a web-based psychoeducational intervention targeting young adults with sexual problems 1.5 years after cancer diagnosis—Results from a randomized controlled trial

**DOI:** 10.1177/20552076241310037

**Published:** 2024-12-26

**Authors:** Charlotta Bergström, Rebecca Skog, Lars E Eriksson, Claudia Lampic, Lena Wettergren

**Affiliations:** 1 Department of Women's and Children's Health, 27106 Karolinska Institutet, Stockholm, Sweden; 2 Department of Surgery and Urology, Danderyd Hospital, Stockholm, Sweden; 3 Department of Public Health and Caring Sciences, 8097 Uppsala University, Uppsala, Sweden; 4 Department of Neurobiology, Care Sciences and Society, 27106 Karolinska Institutet, Stockholm, Sweden; 5 School of Health and Psychological Sciences, City University of London, London, UK; 6 Medical Unit Infectious Diseases, 59562 Karolinska University Hospital, Stockholm, Sweden; 7 Department of Psychology, Umeå University, Umeå, Sweden

**Keywords:** Young adults, psychoeducation, sexual dysfunction, web-based intervention, digital health, randomized controlled trial, cancer

## Abstract

**Objective:**

To test the efficacy of a web-based psychoeducational intervention, Fex-Can Sex, in reducing sexual dysfunction in young adults with cancer.

**Methods:**

This randomized controlled trial evaluated a 12-week web-based self-help intervention. Young adults aged 19–40 who reported sexual dysfunction 1.5 years after cancer diagnosis were drawn from a population-based cohort. Participants were randomized to an intervention group (IG, *n *= 72) or a control group (CG, *n *= 66) that solely received standard care. Primary outcome was assessed by a domain of the Patient Reported Outcome Measures Information Systems^®^ SexFS: “Satisfaction with sex life.” Secondary outcomes included additional SexFS domains, body image (BIS), emotional distress (HADS), health-related quality of life (EORTC QLQ-C30), and self-efficacy related to sex. Surveys were completed at baseline, post-intervention, and three months later. Effects of the intervention were tested with *t*-tests, and linear mixed models (LMMs), including intention-to-treat and subgroup analyses. Additionally, the IG was asked about their experiences of the program with study-specific questions.

**Results:**

There were no differences in primary or secondary outcomes between the IG and the CG at post-intervention. Subgroup analyses showed that individuals with greater sexual problems at baseline improved over time, regardless of group allocation. Participants spent a mean time of 20.7 min on the program. The study-specific items showed that the majority of participants in the IG appreciated the program and would recommend it to others.

**Conclusion:**

The Fex-Can Sex intervention did not show effect on primary and secondary outcomes. Adherence to the intervention was low, and future interventions are recommended to include more interactive components to enhance usage.

**Clinical trial registration:**

The trial was registered on 25 January, 2016 (trial number: 36621459).

## Background

Sexual health is an integral part of life, as acknowledged by the World Health Organization.^1^ Cancer and its treatment may have a negative impact on sexual health. Previous results indicate that among young adults (<40 years) with cancer,^2^ approximately 50% report sexual problems within the first two years after diagnosis,^3^ and a population-based study including only women reported that as many as 68% reported problems.^4^ The reported problems include low interest in sexual activity,^4,5^ vaginal pain,^4^ reduced vaginal lubrication,^4^ and ejaculatory and erectile problems.^6^

According to national^7^ and international^8^ guidelines, healthcare providers should initiate discussions about the possible impact of cancer and its treatment on sexuality with all patients diagnosed with cancer and continue to address the topic throughout the disease trajectory. Access to information about sexual health, including common side effects after cancer treatment and what help is available, may prevent sexual problems and unnecessary concerns. Nevertheless, a recent Swedish population-based study found that approximately 40% of young adults reported that they had not been informed about the potential impact of their cancer treatment on sex life.^9^ Young adults with cancer are therefore at risk of missing out on information and counseling that could alleviate their potential concerns and problems.

Using the internet to search for health information is common among young adults with cancer^10^ and web-based interventions addressing topics of a sensitive nature, such as sexuality, may offer a complement to health care.^11^ For web-based interventions to be effective in reducing problems and increasing well-being, it is recommended that they be based on a theory relevant for the intended outcome^12^ and that information combined with interactive components, including behavior change content, are included.^13,14^

A recent meta-analysis of 11 randomized controlled trials (RCTs) examined the efficacy of internet- and mobile-based psychological interventions targeting sexual dysfunction.^11^ The interventions were based on cognitive behavioral therapy, including for example psychosexual education, mindfulness, relaxation, communication, emotion regulation, homework, and disorder-specific prevention. Findings derived from the meta-analysis demonstrated significantly improved sexual function and satisfaction in women and sexual function in men compared to mostly nonactive control groups. The results from the internet and mobile interventions were found to be comparable to face-to-face interventions, although with smaller effect sizes.^11^ However, most studies had small samples and low adherence to the interventions, which limits generalizability.

Three studies that have evaluated the effects of web-based interventions on sexual dysfunction in people with cancer, of which one^15^ was part of the meta-analysis above,^11^ have to some extent included young adults (<40 years).^15–17^ One RCT that targeted women with breast cancer (*n *= 169)^15^ tested the efficacy of a web-based therapist-guided cognitive behavioral therapy intervention compared to a wait-list control group. Positive effects were demonstrated in favor of the intervention with increased vaginal lubrication, less discomfort during sex, increased sexual pleasure, and improved body image. Schover et al.^16^ tested the efficacy of an internet-based self-help program for women with breast and gynecological cancer (*n *= 58). The self-help program was compared to a similar program with three supplemental individual in-person counseling sessions. The results showed that the participants who received supplemental face-to-face counseling improved significantly in sexual functioning, as compared to the self-help group. Schover et al.^17^ also evaluated another self-help program directed to female patients (*n *= 60), predominantly with breast cancer. It was a pragmatic trial, using a pre–post study design, and positive effects were found on sexual functioning, sexual activity, and use of sexual aids. The results from the few studies that have included young adults suggest that therapist-guided interventions are more effective, but further research is needed to clarify the effects and mechanisms.

In summary, few studies evaluating web-based interventions for sexual dysfunction have included young adults, and to date, no interventions have been developed specifically for the young adult cancer population. This group may experience additional challenges, such as concerns related to dating and being intimate, compared to their older counterparts, who may already have established relationships. The aim of this study was to test the efficacy of the web-based intervention Fertility and Sexuality Following Cancer (Fex-Can) in reducing sexual dysfunction and improving psychosocial outcomes in young adults following a cancer diagnosis compared to standard care.

## Method

### Study design

The Fex-Can project encompasses a national cohort study^18^ with two embedded RCTs, Fex-Can Sex and Fex-Can Fertility, described in detail in a study protocol.^19^ The RCTs include individuals with self-reported fertility-related distress or sexual dysfunction.

The present study evaluated the efficacy of the Fex-Can Sex program in alleviating sexual dysfunction 1.5 years after being diagnosed with cancer. The study was conducted in a two-armed RCT, in which participants were equally randomized to either an intervention or a control arm. The study was reported in accordance with the Consolidated Standards of Reporting Trials (CONSORT) Statements^20,21^ and the TIDieR checklist.^22^

### Sample

**Figure 1.**
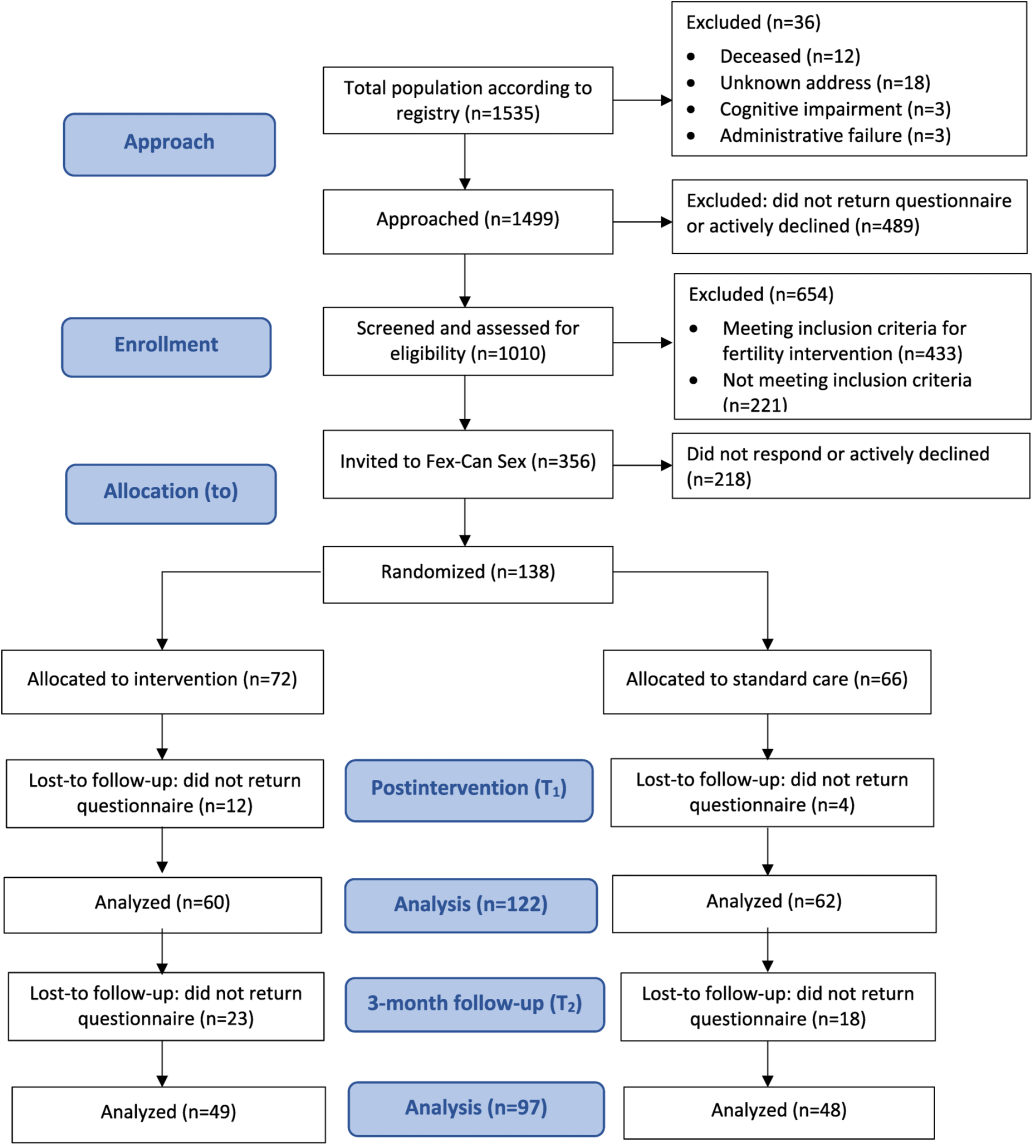
Flow chart.

The sample was drawn from a national cohort study of young adults with selected cancers under the age of 40 at diagnosis. Participants for the cohort study had been identified through Swedish national quality registries for breast, gynecologic, and testicular cancer, lymphoma, and brain tumors. Those in the cohort study who rated sexual dysfunction according to a predefined threshold were invited to participate in the Fex-Can Sex RCT (see Figure 1). The threshold was defined as 0.5 standard deviation (*SD*) from the U.S. population mean in at least one of the selected domains of the Patient Reported Outcome Measures Information Systems (PROMIS®) Sexual Function and Satisfaction Measure (SexFS version 2.0).^19^ Participants who met the cut-off for both fertility-related distress and sexual dysfunction were allocated to one of the interventions by two of the senior researchers according to what problems were most troublesome.

For the Fex-Can Sex program, 356 individuals were approached (271 women and 85 men). Of those approached, 138 returned written consent, a response rate 39%, and were thus included in the RCT. Participants in the RCT were more likely to be female (*p *= .005), have a higher education (*p *= .007), and report ongoing treatment (*p *= .046) compared to non-participants. Further, there was a difference with regard to diagnosis: individuals with breast cancer participated to a higher extent (*p *= .012), whereas those with testicular cancer (*p *= .021) and ovarian cancer (*p *= .013) participated to a lesser extent (see Supplemental Table S1).

### Sample size and allocation

To detect a statistically significant difference with a power of 80% and a medium effect size (0.5), a total of 128 completers of the post-intervention assessment were considered necessary.^19^

Randomization was performed by a statistician, who was not involved in the study, using computer-generated randomization sequences. Participants were randomly assigned to either the intervention group (IG) or the control group (CG) of the Fex-Can Sex with an allocation ratio of 1:1 in blocks stratified by sex and diagnosis. Seventy-two participants were randomized to the IG and 66 to the CG. Blinding of group allocation was not possible for the researchers or participants due to the study design.

### The web-based psychoeducational intervention

The intervention was a 12-week, web-based self-help program, accessible through web browsers on computers, smartphones, and tablets. The intervention was developed in collaboration with patient research partners, former cancer patients diagnosed before the age of 40 years (six women and four men) and two significant others, that is, two mothers of teenagers who had undergone cancer treatment. This was done through a long-term seven-year collaboration with physical meetings one to three times yearly.^23,24^ Details of the intervention, including content and theory, have previously been described in the study protocol^19^ and are presented briefly below.

The intervention was organized in six consecutive modules, with a new module introduced every second week. Each module targeted a specific topic related to sexuality, which is well known to be of relevance for persons diagnosed with cancer. A description of the content of the modules is provided in Table 1. The modules included educational and behavior change content and recorded video vignettes with young adults with the same cancer diagnoses as the study participants. They also included various exercises aiming to, for example, increase body awareness and acceptance and improve sexual function.^23^ The intervention also included a moderated discussion forum, which was shared with participants in the Fex-Can Fertility program. The forum was moderated by one of the patient research partners and a research team member with clinical expertise in nursing or psychology.^25^

**Table 1.** Description of content of the six modules in the Fex-Can Sex program.

Modules	Content		
Sexuality	Introduction to sexuality and cancer-related sex problems with texts and illustrations to describe anatomy and physiology. Expectations about one's sex life, and opportunity for participants to reflect on what sex means to them.	Quotes and portraits, including short video vignettes, by young cancer survivors.	Online moderated discussion forum
Lack of desire	Information about how cancer and its treatment may affect desire. Mindfulness exercises, and on touching and exploring the body with and without a partner. Instructions for pelvic floor exercises.		
Discomfort and pain (women) Erection (men)	Information about how cancer and its treatment may cause pain and discomfort. Presentation of aids helpful in reducing pain. Quiz with questions about the information provided. Exercises to discover what feels good, less good, and uncomfortable, and mindfulness exercises. Information about erection and how cancer and its treatment may impact on erectile ability, physically and psychologically. Brief pharmacological information and medical advice on how to maintain an erection. Exercises and quiz as described for women above.		
Orgasm (women) Orgasm and ejaculation (men)	Information about orgasm and how cancer treatment may affect orgasm ability and pleasure. Exercises relating to orgasm with focus on enhancing pleasure (with and without a partner). Information about orgasm and ejaculation, and on how it may be affected by cancer treatment. Advice for preventing early ejaculation. Exercises as described for women above.		
Relationships and sex	Texts about potential challenges in romantic relationships, dating, and talking to a partner about cancer.		
My body	Information about potential body changes in relation to cancer. Mindfulness exercises aimed at increasing acceptance of a changed body. In this last module, an opportunity to book a consultation with a research team member was included.		

The Fex-Can program is based on self-determination theory^26^ and aims to support the basic psychological needs of autonomy (i.e., skills for managing emotions and relationships), competence (i.e., knowledge and understanding), and relatedness (i.e., social connection to peers and validation of experiences). Information about sexuality following cancer, as well as strategies to manage sexual problems, was included to facilitate participants' sense of autonomy and competence. The discussion forum and video vignettes were included to strengthen participants' relatedness by providing validation of common experiences of sex problems.

### Control group

The CG received standard care, which may or may not have encompassed information and counseling for sex-related problems depending on the diagnosis, treatment, and care setting.

### Data collection

All participants in the RCT completed a survey, including the primary and secondary outcomes at baseline (T0), post-intervention (T1), and three months later (T2). The baseline survey could be completed online or on paper, whereas the follow-up surveys could be completed only on paper. Two reminders were sent to non-responders, and participants received two movie theater gift cards for each completed survey, worth approximately US$20. Participants who did not return post-intervention surveys were considered lost to follow-up. The intervention was carried out in three waves from October 2017 to December 2018. Data collection was completed in June 2019.

*Sociodemographic and clinical data*. Sociodemographic variables included age, sex, country of birth, level of education, occupation, sexual orientation, and partner status and were obtained from the baseline survey (T0) and registries. Clinical data, which included diagnosis, stage, treatment, and relapse, were retrieved from the national quality registries. Based on diagnosis, stage, and treatment regimen, the intensity of each individual's cancer treatment was classified into one of four levels: least, moderate, very, or most, according to the Intensity of Treatment Rating Scale Young Adult.^27^

*Primary outcome.* The primary outcome for the Fex-Can Sex program was the domain “Satisfaction with sex life,” as measured by the PROMIS^®^ SexFS version 2.0.^28^ This domain was chosen as we hypothesized that satisfaction can be improved despite potential long-lasting functional problems. The domain included two items that assessed how pleased the person had been with their sex life, and these were answered on a five-point scale ranging from 1 = *none/not at all* to 5 = *very/a lot*. The scale score was transformed into a T-score metric, where 50 (*SD *= 10) corresponded to the mean of the general U.S. population who had been sexually active in the past 30 days. Sexual dysfunction was defined as 1 *SD*, that is, 10 points on the T-scale, from the population mean of 50. The SexFS has shown adequate content and construct validity, as well as test–retest reliability.^28,29^ The Swedish version of the SexFS has shown satisfactory psychometric properties.^30^

*Secondary outcomes.* Eight domains from the PROMIS^®^ SexFS were used as secondary outcomes.^28^ Three were generic: Interest in sexual activity, Orgasm ability, and Orgasm pleasure, and five were body-part-specific: Vaginal lubrication, Vaginal discomfort, Vulvar discomfort—clitoral, Vulvar discomfort—labial, and Erectile function. Additional secondary outcomes were selected based on factors previously associated with sexual dysfunction in the literature: body image,^5,31^ health-related quality of life (HRQoL),^29,31^ emotional distress,^3^ and self-efficacy.^32^

The Body Image Scale (BIS) assesses body image in relation to cancer, with higher scores reflecting a higher level of body image disturbance. The BIS has shown satisfactory reliability as well as good clinical validity in patients with cancer.^33^ Cronbach's α for the BIS in the current study was .93.

HRQoL was measured with the summary score of the European Organization for the Research and Treatment of Cancer Quality of Life Questionnaire (EORTC QLQ-C30) version 3.0, with higher scores reflecting better HRQoL. The instrument's summary score (based on 13/15 of subscales) has demonstrated robust psychometric properties in cancer populations.^34^ Cronbach's α for the summary score of the QLQ-C30 in the current study was .89.

Emotional distress was assessed using the Hospital Anxiety and Depression Scale (HADS). An overall score was calculated, with higher scores indicating more distress. The HADS has demonstrated satisfactory concurrent validity and internal consistency.^35^ Cronbach's α for the current study was .89.

Sexual self-efficacy, based on Bandura's theory of self-efficacy,^36^ refers to perceived confidence in one's own ability to handle situations, emotions, and thoughts related to sex. Sexual sex efficacy was assessed by six study-specific questions, including, for example, “I feel confident that I can tell when a sexual activity is unpleasant.” Responses were given on a 4-point Likert-type scale ranging from “*not at all*” to “*fully agree*.” A total mean score was calculated, with higher scores indicating greater confidence.

*Self-perceived evaluation of the program.* The postintervention survey at T1 included 13 study-specific items concerning the participants' experiences of the program and were answered by IG participants only. Responses were given on a 4-point Likert scale ranging from “*disagree completely*” to “*agree completely*.” Participants were also asked to rate perceived changes in sexual problems compared to preintervention on a 7-point Likert scale from “*improved a lot*” to “*worsened a lot*.” Two open-ended questions were further included in the postintervention survey. First, participants were given the opportunity to provide comments on potential changes in regard to their sexual problems following the intervention. Second, it was possible to write any additional thoughts about the intervention or about sexuality following cancer.

*Adherence to the intervention.* Adherence to the program was measured with log data retrieved from the website system. Participants were divided into two groups based on their level of adherence to the program: high activity or low activity. High activity was defined as meeting the following criteria: (a) general activity (opened ≥3 modules and spent ≥20 min on the website) and (b) at least one criterion for interactive activity (spent ≥3 min in the discussion forum, wrote ≥1 post, or answered ≥50% of the four reflective questions and the quiz).^37^ All other participants in the IG, including those who had never logged into the program, were defined as low activity users.^37^

### Data analysis

In all tests, an intention-to-treat approach was used to compare the CG and IG. The effects of the intervention, that is, the potential differences between the IG and CG on primary and secondary outcomes, were tested using independent *t*-tests. Cohen's *d* for effect size (ES) was used to interpret clinically important differences between the IG and CG groups. Values of 0.2 were considered small, 0.5 moderate, and >0.8 large.^38^

LMMs with a participant-specific random intercept were used for subgroup analyses. LMMs are widely used to analyze longitudinal data, as the method minimizes the loss of information due to missing scale values.^39^ First, LMMs were used to analyze interaction effects of time and possible changes in sexual function, depending on the participants' baseline levels of sexual dysfunction (high vs. low level of sexual dysfunction). Analyses were conducted for all SexFS domains, with a high level of sexual dysfunction defined as ≥1 *SD* from the U.S. population mean. Second, subgroup analyses were performed by level of activity in the intervention (low level of activity, high level of activity, and CG).

Missing data in the SexFS were handled according to the established PROMIS^®^ methodology.^28^ In the other measures, when single items in the respective scales were missing, the individual's mean was imputed, provided that at least half of the items were answered.

Statistical analyses were conducted using SPSS Statistics version 28 (IBM Corp., Armonk, NY, USA) and the lme4 and lmerTest package in R version 4.2.1. All tests were two-tailed, and a *p*-value of ≤.05 was considered statistically significant.

Responses to open-ended questions included in the postintervention survey were analyzed by one of the authors (RS) using qualitative thematic analysis as described by Braun and Clarke.^40^ In the present study, we focused on the semantic level of meaning in the data in order to stay close to the content of the data and the meaning as expressed by participants.

### Ethical considerations

Written informed consent was collected from all participants. The study was approved by the Regional Board of Ethics in Stockholm (Permit numbers: 2013/1746-31/4, 2014/224-32, and 2017/916-32) and performed according to the ethical standards of the Declaration of Helsinki.

## Results

### Characteristics of participants

A total of 138 individuals were included in the RCT, with 72 randomized to the IG and 66 to the CG (see the flowchart in Figure 1). The sociodemographic and clinical characteristics of the participants are presented in Table 2. The mean age among the participants was 34.3 in the IG and 34.7 in the CG.

**Table 2.** Demographic and clinical characteristics reported at baseline (*N* = 138).

Participant characteristics	Intervention group (*n* = 72)	Control group (*n* = 66)
Sex, *n* (%)		
Women	60 (83)	56 (85)
Men	12 (17)	10 (15)
Country of birth, *n* (%)		
Sweden	65 (90)	59 (89)
Other country	7 (10)	7 (11)
Highest education, *n* (%)		
University	51 (71)	40 (61)
Other education level	21 (29)	26 (39)
Main occupation, *n* (%)		
Working or studying	56 (78)	59 (89)
Unemployed, sick leave, other^a^	16 (22)	7 (11)
Sexual orientation, *n* (%)		
Heterosexual	65 (90)	63 (96)
Nonheterosexual	7 (10)	3 (4)
Relationship status, *n* (%)		
Partnered	53 (74)*	58 (88)
Nonpartnered	19 (26)	8 (12)
Diagnosis, *n* (%)		
Breast cancer	33 (46)	33 (50)
Cervical cancer	17 (24)	16 (24)
Ovarian cancer	1 (1)	0 (0)
Testicular cancer	7 (10)	7 (11)
Lymphoma	9 (12)	6 (9)
Brain tumor	5 (7)	4 (6)
Ongoing treatment (self-reported), *n* (%)		
None	44 (61)	38 (58)
Chemotherapy	4 (6)	3 (4)
Radiation	3 (4)	0 (0)
Hormonal treatment	21 (29)	22 (33)
Other (e.g. antibodies)	6 (8)	4 (6)
Intensity of treatment,^b,c^ *n* (%)		
Least/moderate	26 (37)	25 (40)
Very/most	45 (63)	37 (60)

^a^ Parental leave or retired.

^b^ According to the Intensity of Treatment Rating Young Adult (ITR-YA).

^c^ Does not sum up to total due to missing data.

*Statistically significant difference in proportions between groups (*p* < .05).

The postintervention (T1) survey was completed by 122 participants (88%) and at T2 by 97 participants (70%) (see Figure 1). Responders and non-responders at T1 and T2 did not differ in sociodemographic or clinical characteristics measured at baseline (sex, birth country, education level, occupation status, sexual orientation, partnership, diagnosis, ongoing treatment, and intensity of treatment).

Among the study participants (IG and CG), baseline levels of sexual dysfunction did not differ between responders and non-responders at the follow-ups. However, non-responders at T1 reported lower HRQoL at baseline as assessed by the EORTC QLQ-C30 (65.9 vs. 74.7, *t *= ‒1.982; *p *= .050) as well as more emotional distress as measured with HADS (19.7 vs. 12.9, *t *= 3.641; *p *< .001). Responders at T2 were, to a higher extent, on hormonal treatment at baseline (37% vs. 17%, χ^2 ^= 5.396; *p *= .020).

### Activity in the intervention

The majority of IG participants were categorized as low-activity users, which included those who never opened the program (*n *= 5). Only 16/72 (22%) of the participants in the IG met the criteria for high activity usage. In terms of the mean, participants spent 20.7 (*SD *= 21.3) minutes in total in the program, which did not differ between women and men. About half (40/72, 56%) of the participants opened three modules or more, and about one in four (19/72, 24%) responded to at least 50% of the reflective questions and the quiz. While a majority of participants in the IG spent time in the discussion forum, with a mean time of 4.0 (*SD *= 9.0) minutes, only seven wrote their own posts.

### Effects of the intervention

*Primary and secondary outcomes.* No statistically significant differences for the primary or secondary outcomes were found between the IG and CG post-intervention (T1) or three months later (T2) (see Table 3). No notable effect sizes were seen on the primary or secondary outcomes (data not shown).

**Table 3.** Comparisons of mean scores of primary (“Satisfaction with sex life”) and secondary outcomes between the intervention group (IG) and the control group (CG), with an intention-to-treat approach, at baseline, post-intervention, and three-month follow-up.

Outcomes	T0 (baseline)		T1 (post-intervention)			T2 (3-months follow-up)		
	IG (*n* = 72) Mean (SD)	CG (*n* = 66) Mean (SD)	IG (*n* = 60) Mean (SD)	CG (*n* = 62) Mean (SD)	*p*	IG (*n* = 49) Mean (SD)	CG (*n* = 48) Mean (SD)	*p*
Satisfaction with sex life^a,↑^	43.70 (7.64) *n* = 70	42.43 (7.53) *n* = 60	45.22 (7.02) *n* = 56	44.50 (6.32) *n* = 55	.573^d^	45.88 (6.98) *n* = 42	43.96 (7.01) *n* = 40	.217^e^
Interest in sexual activity^a,↑^	41.06 (9.87) *n* = 72	39.74 (9.75) *N* = 65	40.04 (9.17) *n* = 60	40.37 (10.89) *n* = 61	.860	39.74 (10.18) *n* = 49	40.10 (10.92) *n* = 48	.865
Orgasm—ability^a,↑^	46.15 (11.77) *n* = 66	43.26 (11.97) *n* = 59	49.55 (10.02) *n* = 51	46.56 (10.36) *n* = 54	.136	46.71 (10.88) *n* = 41	50.04 (10.49) *n* = 36	.176
Orgasm—pleasure^a,↑^	47.19 (8.71) *n* = 63	46.12 (9.10) *n* = 56	48.20 (6.97) *n* = 49	47.78 (8.29) *n* = 51	.782	46.41 (8.55) *n* = 40	48.33 (8.79) *n* = 36	.338
Vaginal lubrication^a,↑^	42.59 (8.50) *n* = 58	42.04 (9.84) *n* = 49	41.99 (10.19) *n* = 47	42.97 (8.98) *n* = 46	.622	43.23 (9.57) *n* = 34	44.16 (9.17) *n* = 31	.692
Vaginal discomfort^a,↓^	58.28 (8.98) *n* = 55	58.14 (9.58) *n* = 48	56.21 (10.06) *n* = 44	54.49 (9.41) *n* = 45	.407	56.98 (10.40) *n* = 31	54.24 (9.97) *n* = 31	.294
Vulvar discomfort—clitoral^a,↓^	57.39 (10.30) *n* = 57	54.73 (8.84) *n* = 49	55.47 (10.28) *n* = 48	52.67 (8.05) *n* = 45	.147	56.02 (9.53) *n* = 33	52.01 (8.16) *n* = 31	.076
Vulvar discomfort—labial^a,↓^	57.83 (10.13) *n* = 57	56.37 (8.80) *n* = 49	55.52 (9.80) *n* = 48	54.00 (8.41) *n* = 45	0.426	54.66 (9.89) *n* = 34	53.26 (8.22) *n* = 31	.537
Erectile function^a,c,↑^	41.52 (6.97) *n* = 12	48.32 (8.31) *n* = 10	46.94 (7.51) *n* = 8	49.37 (10.00) *n* = 9	.184	45.87 (5.23) *n* = 8	48.86 (9.94) *n* = 7	.352
Body Image Scale^↓^	11.49 (7.43) *n* = 72	11.47 (6.98) *n* = 66	10.08 (6.05) *n* = 60	10.93 (6.62) *n* = 61	.465	9.18 (6.26) *n* = 49	10.04 (6.88) *n* = 48	.522
Hospital Anxiety Depression Scale^↓^	15.01 (7.55) *n* = 72	12.25 (6.96) *n* = 66	13.90 (7.33) *n* = 59	12.40 (6.96) *n* = 62	.252	13.59 (8.53) *n* = 49	11.41 (7.33) *n* = 48	.180
EORTC QLQ-C30^b,↑^	72.47 (16.54) *n* = 72	75.01 (17.30) *n* = 65	75.45 (16.10) *n* = 60	78.62 (14.76) *n* = 62	.258	77.64 (14.61) *n* = 49	78.37 (17.92) *n* = 48	.825
Self-efficacy sex^↑^	3.16 (0.67) *n* = 69	3.22 (0.59) *n* = 66	3.31 (0.60) *n* = 56	3.26 (0.58) *n* = 60	.702	3.19 (0.69) *n* = 45	3.29 (0.59) *n* = 47	.448

*Note:* EORTC QLQ-C30: European Organization for the Research and Treatment of Cancer Quality of Life Questionnaire.

The number of observations differs between domains due because domains are gender-specific.

^a^ PROMIS SexFS domains.

^b^ EORTC Quality of Life Core Questionnaire.

^c^ Mann–Whitney U-test.

^d^ Cohen's *d *= 0.107.

^e^ Cohen's *d *= 0.275.

^↑^ Higher scores indicate better sexual function, better health-related quality of life, and higher level of self-efficacy related to sex.

^↓^ Lower scores indicate better sexual function, less anxiety and depression, and a more positive body image.

*Subgroup analyses.* The subgroup analyses showed that participants with high levels of sexual dysfunction at baseline (≥1 *SD*), in both the IG and the CG, improved over time in several SexFS domains. At T1, the IG had improved in six domains: Vulvar discomfort—clitoral (*p *< .001), Vulvar discomfort—labial (*p *< .001), Interest in sexual activity (*p *= .001), Satisfaction with sex life (*p *= .017), Orgasm ability (*p *= .035), and Orgasm pleasure (*p *< .001). The CG had improved in seven of the domains at T1: Vaginal discomfort (*p *= .003), Vulvar discomfort—clitoral (*p *< .001), Vulvar discomfort—labial (*p *< .001), Interest in sexual activity (*p *= .012), Satisfaction with sex life (*p *< .001), Orgasm ability (*p *< .001), and Orgasm pleasure (*p *= .013). At T2, the IG had statistically improved in eight of the domains and the CG in five; for more details (see supplemental Figures A‒R). No corresponding change was found in the groups that reported lower levels of sexual dysfunction at baseline.

Further, subgroup analyses showed that high activity in the program did not change the results (data not shown).

*Self-perceived evaluation of the program.* At postintervention, items to assess participants' experiences of the program were completed by 56/72 (78%) participants from the IG. The majority of responders indicated that they appreciated the content of the program 41/56 (73%) and noted that they would recommend it to others 51/56 (91%). Further, 35/56 (63%) reported that the program had helped them to deal with their problems, and 10/56 (18%) reported that their sexual problems had improved or improved a lot.

Additionally, there were two open-ended questions posed in the post-intervention survey. The first covered whether participants experienced changes in sexual problems compared to before taking part in the program, and lastly participants were offered the opportunity to add any additional thoughts about the program or sexuality. The questions were responded to by 18/56 (32%) and 27/56 (48%) participants, respectively. Four main themes were constructed in the thematic analysis: “Perceived benefits,” “Barriers to participation,” “Not for me,” and “Suggestions for the future.”

*Perceived benefits.* The main benefits described by participants were increased acceptance and relatedness. This included an increased acceptance of sexual problems as well as of one's life situation and self in general. Many recognized themselves in the content included in the program and appreciated knowing that they were not alone. Finally, some participants expressed an increased frequency of communication about problems with their partners following the program.

*Barriers to participation.* Time constraints were identified as a barrier to participation. Participants expressed that competing responsibilities and insufficient time hindered their ability to take part in the program to the extent that they would have liked.

*Not for me.* Some participants felt that the program was not for them for a variety of reasons. The reasons included not being in direct need of the program, feeling that their diagnosis (e.g., brain tumor) was not adequately represented in the program, and experiencing the program as a reminder of cancer.

*Suggestions for the future.* A number of wishes concerning the program were also expressed. More information and exercises for the partner, as well as a diagnosis-specific program, were suggested. Continued access to the program, including the discussion forum was mentioned. Finally, participants wished for fertility-related information to be included.

## Discussion

This RCT evaluated the efficacy of a web-based psychoeducational self-help intervention for alleviating sexual dysfunction in young adults who had been diagnosed with cancer approximately 1.5 years earlier. No statistically significant differences in primary or secondary outcomes were observed between the IG and CG post-intervention. However, subgroup analyses revealed that sexual function improved over time for those who indicated more sexual problems at baseline, regardless of group allocation.

In contrast to our self-help intervention, results from other web-based interventions with more guidance have demonstrated significant improvements in a number of outcomes. Hummel et al.^15^ evaluated a therapist-guided intervention and reported significantly better overall sexual function and body image in the IG compared to the CG. Moreover, Schover et al.^16^ found significantly better results in sexual function and satisfaction when a self-help program was used with guidance rather than alone.^16^ In the self-guided group, no significant improvement post-intervention was found in any of the sexual outcomes. However, the group improved in terms of emotional distress and quality of life.

We hypothesized that the intervention would cause proximal outcomes (including increased knowledge, social connectedness, and skills), which in turn would support the participants' basic psychological needs before affecting any long-term outcomes.^13^ Self-efficacy is assumed to be a proxy measure for competence,^41^ encompassing perceived confidence in one's ability to successfully perform certain tasks.^42^ Previous research has indicated that low sexual self-efficacy is associated with more sexual problems.^32^ Future studies are recommended to also assess satisfaction with basic psychological needs to gain a deeper understanding of the underlying mechanisms of the results.

Low adherence to digital interventions is a commonly reported problem.^43^ As young adults are likely to be in the middle of a career and/or family building, their time available for attending an intervention may be limited,^44^ which was also confirmed in comments in the postintervention survey. In our study, only 16/72 (22%) of the IG participants fulfilled the criteria for high activity use, and on average, participants only spent 21 min on the intervention program. Micaux et al.^37^ reported on the efficacy of the Fex-Can Fertility program and found similar challenges with adherence, with 33% of the participants in their study fulfilling the criteria for high activity use. An interview study conducted after the RCT with participants from the current study explored experiences of participating in the intervention.^44^ The described practical obstacles of engaging in the program included perceived lack of time and inconvenient timing for the intervention. While we did not define the amount of time or level of activity that would be necessary for participants to benefit from the intervention a priori, it can be hypothesized that the amount spent was not enough to achieve significant improvement. Hummel et al.^15^ estimated that participants in their intervention needed to complete a minimum of five sessions, each taking approximately 90–120 min, in order to expect any appreciable effect.^45^ Their intervention included weekly contact with a therapist, which encouraged attendance and participation, and reminders were sent to participants when needed. The intervention was successfully completed (according to the judgment of the therapist) by 62% of the participants. Participants in previous self-guided interventions spent more time on the interventions than participants in our study did, on average 82^17^‒109^16^ min, respectively. Some differences between previous self-guided interventions and the Fex-Can can be noted. One of the previous self-help interventions used a self-recruiting sampling strategy,^17^ and none of the two studies used pre-defined cut-offs for sexual dysfunction; rather, self-perceived sex problems were used as inclusion criteria. The pragmatic trial by Schover et al. 2020^17^ was more individualized and included goal-setting features. Another study including young individuals with cancer who struggled with adherence was a feasibility study by Classen et al.^46^ which evaluated an online support group consisting of a discussion forum. A first evaluation showed that the time spent in the intervention was low, and a 30-min individual introduction was therefore added before conducting the intervention for participants in the wait-listed group. This change almost doubled the participation rates as compared to the first implementation round.^46^ Thus, digital interventions seem to benefit from therapeutic guidance, individualization, and introduction.

Explanations for the low usage of the program in the present study are likely to be multifactorial. First, we believe the inclusion criterion for sexual dysfunction (0.5 *SD* from the population mean) was too low to include only those most in need of such support. If participants do not perceive their sexual problems as particularly distressing or bothersome, their motivation for program use may be low, and there might be relatively little room for improvement. In contrast, Schover et al.^17^ included individuals with considerably higher levels of sexual dysfunction at baseline, as much as 10 points above ours, and the participants reported statistically significant improvements in several domains. Second, due to the self-guided and untailored nature of our program, all participants received the same set of modules, and no feedback was given on, for example, completed exercises. Although all participants were offered a phone call or meeting at the start and end of the intervention, only a few accepted that offer. While self-guided interventions are cost-effective^47^ and can provide participants with timely support and information, we know from previous literature that additional guidance or tailored content may be a beneficial feature to include in internet interventions.^48^ These findings, that web-based unguided interventions suffer from poorer adherence than those that are guided, are consistent with the literature.^48^ An explanation is that guided support may increase motivation to participate.^49^ Future research should investigate the role of different technologies (e.g. mobile notifications) and the level of guidance and feedback to enhance user engagement. Third, the intervention components included in the present study may need to be revised. For example, some reported them to be too generalized and stated that they wanted more individualized advice, for example, specific information targeted to individuals with brain tumors, and others reported the length of the texts to be too long and extensive and expressed the desire for more audiofiles.^44^

### Methodological considerations

Strengths of our study include the design, the theory-based intervention^26^ developed with a participatory research approach,^23^ and use of validated instruments. Further, response rates were good at follow-ups, although non-responders at follow-up were more likely to have reported a lower quality of life and more emotional distress at baseline than the responders. However, the study also has limitations. RCTs are generally considered to be the gold standard in scientific research. Nevertheless, in social and psychological interventions, particularly digital interventions, conditions are not entirely controlled, as double blinding is not possible. We did not query the participants as to whether they sought any other help or support for their sexual problems during the time of the RCT. Also, the low participation rate among men, which is in line with previous studies,^17^ made it difficult to draw firm conclusions about the intervention's effect on men. Similar difficulties with recruitment were found in one of the studies by Schover et al.,^17^ resulting in exclusion of the men in the analyses, with results presented for women only. Low recruitment and participation rates of men in health behavior research have been reported previously,^50^ and women are more likely to enroll^51^ and adhere^49^ to online psychological interventions. More research is needed to investigate the support and format preferences of men and how to encourage men to participate in digital interventions.

## Conclusion

The Fex-Can Sex web-based intervention did not yield statistically significant effects on primary or secondary outcomes. Despite the lack of effect, participants appreciated the program, and some reported it to be helpful. Usage and activity in the intervention were limited, which is likely to have contributed to the absence of the predicted outcomes. Based on our results, the intervention is being revised, and the new version, Fex-Can 2.0, will take greater account of the extent of perceived problems, and participants will be recommended content based on their individual needs. Additionally, Fex-Can 2.0 will include more interactive components to enhance adherence and activity, including, for example, increased guidance and feedback from research team members.

## Supplemental Material

sj-docx-1-dhj-10.1177_20552076241310037 - Supplemental material for Efficacy of a web-based psychoeducational intervention targeting young adults with sexual problems 1.5 years after cancer diagnosis—Results from a randomized controlled trial Supplemental material, sj-docx-1-dhj-10.1177_20552076241310037 for Efficacy of a web-based psychoeducational intervention targeting young adults with sexual problems 1.5 years after cancer diagnosis—Results from a randomized controlled trial by Charlotta Bergström, Rebecca Skog, Lars E Eriksson, Claudia Lampic and Lena Wettergren in DIGITAL HEALTH

sj-docx-2-dhj-10.1177_20552076241310037 - Supplemental material for Efficacy of a web-based psychoeducational intervention targeting young adults with sexual problems 1.5 years after cancer diagnosis—Results from a randomized controlled trial Supplemental material, sj-docx-2-dhj-10.1177_20552076241310037 for Efficacy of a web-based psychoeducational intervention targeting young adults with sexual problems 1.5 years after cancer diagnosis—Results from a randomized controlled trial by Charlotta Bergström, Rebecca Skog, Lars E Eriksson, Claudia Lampic and Lena Wettergren in DIGITAL HEALTH

sj-docx-3-dhj-10.1177_20552076241310037 - Supplemental material for Efficacy of a web-based psychoeducational intervention targeting young adults with sexual problems 1.5 years after cancer diagnosis—Results from a randomized controlled trial Supplemental material, sj-docx-3-dhj-10.1177_20552076241310037 for Efficacy of a web-based psychoeducational intervention targeting young adults with sexual problems 1.5 years after cancer diagnosis—Results from a randomized controlled trial by Charlotta Bergström, Rebecca Skog, Lars E Eriksson, Claudia Lampic and Lena Wettergren in DIGITAL HEALTH

## References

[1] World Health Organization. World Health Organization: sexual health, https://www.who.int/health-topics/sexual-health#tab=tab_2 (2006, accessed 24 November 2024).

[2] Aubin S Barr R Rogers P , et al. What should the age range be for AYA oncology? J Adolesc Young Adult Oncol 2011; 1: 3–10. DOI: 10.1089/jayao.2011.1505. 26812562

[3] Acquati C Zebrack BJ Faul AC , et al. Sexual functioning among young adult cancer patients: a 2-year longitudinal study. Cancer 2018; 124: 398–405. DOI: 10.1002/cncr.31030. 29149503 10.1002/cncr.31030 PMC7514897

[4] Wettergren L Eriksson LE Bergström C , et al. Prevalence and risk factors for sexual dysfunction in young women following a cancer diagnosis – a population-based study. Acta Oncol 2022; 61: 1165–1172. DOI: 10.1080/0284186X.2022.2112283. 36176069

[5] Ljungman L Eriksson LE Flynn KE , et al. Sexual dysfunction and reproductive concerns in young men diagnosed with testicular cancer: an observational study. J Sex Med 2019; 16: 1049–1059. DOI: 10.1016/j.jsxm.2019.05.005. 31255211 10.1016/j.jsxm.2019.05.005

[6] Sanchez Varela V Zhou ES Bober SL . Management of sexual problems in cancer patients and survivors. J Sex Med 2013; 37: 319–352. DOI: 10.1016/j.currproblcancer.2013.10.009. 10.1016/j.currproblcancer.2013.10.009 24331239

[7] Confederation of Regional Cancer Centers. Cancer rehabilitering, Nationellt vårdprogram 2023, https://kunskapsbanken.cancercentrum.se/globalassets/vara-uppdrag/rehabilitering-palliativ-vard/vardprogram/nationellt-vardprogram-cancerrehabilitering.pdf (2023, accessed 31 May 2024).

[8] Carter J Lacchetti C Andersen BL , et al. Interventions to address sexual problems in people with cancer: American Society of Clinical Oncology clinical practice guideline adaptation of Cancer Care Ontario guideline. J Clin Oncolinical Oncology 2018; 36: 492–511. DOI: 10.1200/jco.2017.75.8995. 10.1200/JCO.2017.75.8995 29227723

[9] Bergström C Lampic C Roy R , et al. Do young adults with cancer receive information about treatment-related impact on sex life? Results from a population-based study. Cancer Med 2023; 12: 9893–9901. DOI: 10.1002/cam4.5672. 36748659 PMC10166940

[10] Mooney R Samhouri M Holton A , et al. Adolescent and young adult cancer survivors’ perspectives on their internet use for seeking information on healthy eating and exercise. J Adolesc Young Adult Oncol 2017; 6: 367–371. DOI: 10.1089/jayao.2016.0059. 27845844 10.1089/jayao.2016.0059 PMC5467135

[11] Zarski AC Velten J Knauer J , et al. Internet- and mobile-based psychological interventions for sexual dysfunctions: a systematic review and meta-analysis. NPJ Digit Med 2022; 5: 139. DOI: 10.1038/s41746-022-00670-1. 36085306 10.1038/s41746-022-00670-1 PMC9463146

[12] Fernandez ME Ruiter RAC Markham CM , et al. Intervention mapping: theory- and evidence-based health promotion program planning: perspective and examples. Front Public Health 2019; 7: 209. DOI: 10.3389/fpubh.2019.00209. 31475126 10.3389/fpubh.2019.00209 PMC6702459

[13] Pingree S Hawkins R Baker T , et al. The value of theory for enhancing and understanding e-health interventions. Am J Prev Med 2010; 38: 103–109. DOI: 10.1016/j.amepre.2009.09.035. 20117565 10.1016/j.amepre.2009.09.035 PMC2826889

[14] Barak A Klein B Proudfoot JG . Defining internet-supported therapeutic interventions. Ann Behav Med 2009; 38: 4–17. DOI: 10.1007/s12160-009-9130-7. 19787305 10.1007/s12160-009-9130-7

[15] Hummel SB van Lankveld JJDM Oldenburg HSA , et al. Efficacy of internet-based cognitive behavioral therapy in improving sexual functioning of breast cancer survivors: results of a randomized controlled trial. J Clin Oncol 2017; 35: 1328–1340. DOI: 10.1200/jco.2016.69.6021. 28240966 10.1200/JCO.2016.69.6021

[16] Schover LR Yuan Y Fellman BM , et al. Efficacy trial of an internet-based intervention for cancer-related female sexual dysfunction. J Natl Compr Canc Netw 2013; 11: 1389–1397. DOI: 10.6004/jnccn.2013.0162. 24225972 10.6004/jnccn.2013.0162 PMC3831175

[17] Schover LR Strollo S Stein K , et al. Effectiveness trial of an online self-help intervention for sexual problems after cancer. J Sex Marital Ther 2020; 46: 576–588. DOI: 10.1080/0092623x.2020.1762813. 32400321 10.1080/0092623X.2020.1762813

[18] Wettergren L Ljungman L Obol CM , et al. Sexual dysfunction and fertility-related distress in young adults with cancer over 5 years following diagnosis: study protocol of the Fex-Can cohort study. BMC Cancer 2020; 20: 9. DOI: 10.1186/s12885-020-07175-8. 32758179 10.1186/s12885-020-07175-8 PMC7409491

[19] Lampic C Ljungman L Micaux Obol C , et al. A web-based psycho-educational intervention (Fex-Can) targeting sexual dysfunction and fertility-related distress in young adults with cancer: study protocol of a randomized controlled trial. BMC Cancer 2019; 19: 344. DOI: 10.1186/s12885-019-5518-3. 30975116 10.1186/s12885-019-5518-3 PMC6458789

[20] Montgomery P Grant S Mayo-Wilson E , et al. Reporting randomised trials of social and psychological interventions: the CONSORT-SPI 2018 extension. Trials 2018; 19: 407. DOI: 10.1186/s13063-018-2733-1. 30060754 10.1186/s13063-018-2733-1 PMC6066921

[21] Eysenbach G . CONSORT-EHEALTH: improving and standardizing evaluation reports of web-based and mobile health interventions. J Med Internet Res 2011; 13: e126. DOI: 10.2196/jmir.1923. 10.2196/jmir.1923 PMC3278112 22209829

[22] Hoffmann TC Glasziou PP Boutron I , et al. Better reporting of interventions: template for intervention description and replication (TIDieR) checklist and guide. BMJ (Clinical Res Ed) 2014; 348: g1687. DOI: 10.1136/bmj.g1687. 10.1136/bmj.g1687 24609605

[23] Winterling J Wiklander M Obol CM , et al. Development of a self-help web-based intervention targeting young cancer patients with sexual problems and fertility distress in collaboration with patient research partners. JMIR Res Protoc 2016; 5: e60. DOI: 10.2196/resprot.5499. 10.2196/resprot.5499 PMC4846786 27073057

[24] Hovén E Eriksson L Månsson D'Souza Å , et al. What makes it work? Exploring experiences of patient research partners and researchers involved in a long-term co-creative research collaboration. Res Involv Engagem 2020; 6: 33. DOI: 10.1186/s40900-020-00207-4. 32579132 10.1186/s40900-020-00207-4 PMC7305606

[25] Skog R Lampic C Olsson E , et al. The role of a discussion forum within a web-based psychoeducational intervention focusing on sex and fertility-What do young adults communicate? Cancer Med 2023; 12: 17273–17283. DOI: 10.1002/cam4.6317. 37401398 PMC10501254

[26] Ryan RM Deci EL . Self-determination theory and the facilitation of intrinsic motivation, social development, and well-being. Am Psychol 2000; 55: 68–78. DOI: 10.1037//0003-066x.55.1.68. 11392867 10.1037//0003-066x.55.1.68

[27] Hedman C Ahlgren J Smedby KE , et al. Cancer in young adulthood - classifying the intensity of treatment. Acta Oncol 2022; 61: 809–813. DOI: 10.1080/0284186x.2022.2071110. 35575147 10.1080/0284186X.2022.2071110

[28] Weinfurt KP Lin L Bruner DW , et al. Development and initial validation of the PROMIS (®) sexual function and satisfaction measures version 2.0. J Sex Med 2015; 12: 1961–1974. DOI: 10.1111/jsm.12966. 26346418 10.1111/jsm.12966

[29] Flynn KE Jeffery DD Keefe FJ , et al. Sexual functioning along the cancer continuum: focus group results from the patient-reported outcomes measurement information system (PROMIS®). Psychooncology 2011; 20: 378–386. DOI: 10.1002/pon.1738. 20878833 10.1002/pon.1738 PMC3013236

[30] Hovén E Flynn KE Weinfurt KP , et al. Psychometric evaluation of the Swedish version of the PROMIS sexual function and satisfaction measures in clinical and nonclinical young adult populations. Sex Med 2023; 11: 1–9. DOI:10.1093/sexmed/qfac006. PMC10065179 37007849

[31] Ljungman L Ahlgren J Petersson LM , et al. Sexual dysfunction and reproductive concerns in young women with breast cancer: type, prevalence, and predictors of problems. Psychooncology 2018; 27: 2770–2777. DOI: 10.1002/pon.4886. 30203884 10.1002/pon.4886 PMC6585728

[32] Hungr C Recklitis CJ Wright AA , et al. How does a single session group intervention improve sexual function in ovarian cancer survivors? A secondary analysis of effects of self-efficacy, knowledge and emotional distress. Psychol Health Med 2020; 25: 110–120. DOI: 10.1080/13548506.2019.1626452. 31167551 10.1080/13548506.2019.1626452

[33] Hopwood P Fletcher I Lee A , et al. A body image scale for use with cancer patients. Eur J Cancer 2001; 37: 189–197. DOI: 10.1016/s0959-8049(00)00353-1. 11166145 10.1016/s0959-8049(00)00353-1

[34] Giesinger JM Kieffer JM Fayers PM , et al. Replication and validation of higher order models demonstrated that a summary score for the EORTC QLQ-C30 is robust. J Clin Epidemiol 2016; 69: 79–88. DOI: 10.1016/j.jclinepi.2015.08.007. 26327487 10.1016/j.jclinepi.2015.08.007

[35] Bjelland I Dahl AA Haug TT , et al. The validity of the hospital anxiety and depression scale. An updated literature review. J Psychosom Res 2002; 52: 69–77. DOI: 10.1016/s0022-3999(01)00296-3. 11832252 10.1016/s0022-3999(01)00296-3

[36] Bandura A . Self-efficacy: the exercise of control. New York: W H Freeman/Times Books/Henry Holt & Co, 1997.

[37] Micaux C Wiklander M Eriksson LE , et al. Efficacy of a web-based psychoeducational intervention for young adults with fertility-related distress following cancer (Fex-Can): randomized controlled trial. JMIR Cancer 2022; 8: e33239. DOI: 10.2196/33239. 10.2196/33239 PMC9006131 35348459

[38] Cohen J Hillsdale L . Statistical power analysis for the behavioral sciences. Hillsdale, NY: Erlbaum Associates, 1988.

[39] Hesser H . Modeling individual differences in randomized experiments using growth models: recommendations for design, statistical analysis and reporting of results of internet interventions. Internet Interv 2015; 2: 110–120. DOI: 10.1016/j.invent.2015.02.003.

[40] Braun V Clarke V . Research designs: quantitative, qualitative, neuropsychological, and biological. APA handbook of Research Methods in Psychology 2012; 2: 57–71.

[41] Ryan R Deci E . Self-determination theory: basic psychological needs in motivation, development, and wellness. New York: The Guildford Press, 2018: 378‒381.

[42] Bandura A . Self-efficacy: toward a unifying theory of behavioral change. Psychol Rev 1977; 84: 191–215. DOI: 10.1037//0033-295x.84.2.191. 847061 10.1037//0033-295x.84.2.191

[43] Sieverink F Kelders SM van Gemert-Pijnen JEWC . Clarifying the concept of adherence to ehealth technology: systematic review on when usage becomes adherence. J Med Internet Res 2017; 19: e402. DOI: 10.2196/jmir.8578. 10.2196/jmir.8578 PMC5738543 29212630

[44] Obol CM Lampic C Wettergren L , et al. Experiences of a web-based psycho-educational intervention targeting sexual dysfunction and fertility distress in young adults with cancer—a self-determination theory perspective. PloS One 2020; 15: 16. DOI: 10.1371/journal.pone.0236180. 10.1371/journal.pone.0236180 PMC7375532 32697801

[45] Hummel SB van Lankveld JJDM Oldenburg HS , et al. Internet-based cognitive behavioral therapy for sexual dysfunctions in women treated for breast cancer: design of a multicenter, randomized controlled trial. BMC Cancer 2015; 15: 321. DOI: 10.1186/s12885-015-1320-z. 25927495 10.1186/s12885-015-1320-z PMC4423483

[46] Classen CC Chivers ML Urowitz S , et al. Psychosexual distress in women with gynecologic cancer: a feasibility study of an online support group. Psychooncology 2013; 22: 930–935. DOI: 10.1002/pon.3058. 22374732 10.1002/pon.3058

[47] Beatty L Lambert S . A systematic review of internet-based self-help therapeutic interventions to improve distress and disease-control among adults with chronic health conditions. Clin Psychol Rev 2013; 33: 609–622. DOI: 10.1016/j.cpr.2013.03.004. 23603521 10.1016/j.cpr.2013.03.004

[48] Baumeister H Reichler L Munzinger M , et al. The impact of guidance on internet-based mental health interventions — a systematic review. Internet Interv 2014; 1: 205–215. DOI: 10.1016/j.invent.2014.08.003.

[49] Beatty L Binnion C . A systematic review of predictors of, and reasons for, adherence to online psychological interventions. Int J Behav Med 2016; 23: 776–794. DOI: 10.1007/s12529-016-9556-9. 26957109 10.1007/s12529-016-9556-9

[50] Ryan J Lopian L Le B , et al. It’s not raining men: a mixed-methods study investigating methods of improving male recruitment to health behaviour research. BMC Public Health 2019; 19: 814. DOI: 10.1186/s12889-019-7087-4. 31234825 10.1186/s12889-019-7087-4 PMC6591998

[51] Beatty L Kemp E Binnion C , et al. Uptake and adherence to an online intervention for cancer-related distress: older age is not a barrier to adherence but may be a barrier to uptake. Support Care Cancer 2017; 25: 1905–1914. DOI: 10.1007/s00520-017-3591-1. 28155018 10.1007/s00520-017-3591-1

