# Supplementary material for: Efficacy of a web-based psychoeducational intervention targeting young adults with sexual problems 1.5 years after cancer diagnosis—Results from a randomized controlled trial
Source: Digit Health. 2024 Dec 26;10:20552076241310037. doi: 10.1177/20552076241310037 (PMC11686631; doi:10.1177/20552076241310037)
Supplement: sj-docx-1-dhj-10.1177_20552076241310037 - Supplemental material for Efficacy of a web-based psychoeducational intervention targeting young adults with sexual problems 1.5 years after cancer diagnosis—Results from a randomized controlled trial [file sj-docx-1-dhj-10.1177_20552076241310037.docx]

| **Characteristics** | **RCT** **participants**  n=138 | **Non-participants RCT** n=218 | **p-value** |
| --- | --- | --- | --- |
| **Sex, n (%)**  Women  Men |  |  | **0,005** |
|  | 116 (84) | 155 (71) |  |
|  | 22 (16) | 63 (29) |  |
| **Age (years)**, mean (SD) / median, range | 34.5 (4.8) | 34.4 (4.8) | 0.886 |
| **Country of birth, n (%)**  Sweden  Other country |  |  | 0.373 |
|  | 124 (90) | 189 (87) |  |
|  | 14 (10) | 29 (13) |  |
| **Highest education, n (%)**  University  Other education level |  |  | **0.007** |
|  | 91 (66) | 112 (51) |  |
|  | 47 (34) | 106 (49) |  |
| **Main occupation, n (%)**  Working or studying  Unemployed, sick-leave, other^a^ |  |  | 0.184 |
|  | 115 (83) | 169 (78) |  |
|  | 23 (17) | 49 (22) |  |
| **Sexual orientation, n (%)**  Heterosexual  Non-heterosexual |  |  | 0.807 |
|  | 128 (93) | 199 (93) |  |
|  | 10 (7) | 14 (7) |  |
| **Relationship status, n (%)**  Partnered  Non-partnered |  |  | 0.333 |
|  | 111 (80) | 184 (84) |  |
|  | 27 (20) | 34 (16) |  |
| **Diagnosis,^b,c^ n (%)**  Breast cancer  Cervical cancer  Ovarian cancer  Testicular cancer  Lymphoma  Brain tumor |  |  |  |
|  | 66 (48) | 75 (34) | **0.012** |
|  | 33 (24) | 43 (20) | 0.347 |
|  | 1 (1) | 13 (6) | **0.013** |
|  | 14 (10) | 42 (19) | **0.021** |
|  | 15 (11) | 23 (11) | 0.924 |
|  | 9 (6) | 22 (10) | 0.244 |
| **Ongoing treatment (self-reported),^b^ n (%)**  Off treatment  On treatment  Chemotherapy  Radiation  Hormonal treatment  Other (eg. antibodies) |  |  | **0.046** |
|  | 82 (59) | 150 (69) |  |
|  | 56 (41) | 65 (30) |  |
|  | 7 (5) | 12 (6) |  |
|  | 3 (2) | 5 (2) |  |
|  | 43 (31) | 49 (23) |  |
|  | 10 (7) | 7 (3) |  |
| **Intensity of treatment,^d,e^ n (%)**  Least/moderate  Very/most |  |  | 0.89 |
|  | 51 (38) | 102 (48) |  |
|  | 82 (62) | 112 (52) |  |
| ^a^Parental leave or retired.  ^b^Chi-square computation was based only on valid cases: df = 1.  ^c^Each diagnosis tested against all other diagnoses  ^d^According to the Intensity of Treatment Rating Young Adult (ITR-YA).  ^e^Does not sum up to total due to missing data. | | |  |

**Supplementary Table 1.** Demographic and clinical characteristics of eligible participants for the study, RCT participants and non-participants, at T0 (baseline)
