# Supplementary material for: Efficacy of a web-based psychoeducational intervention targeting young adults with sexual problems 1.5 years after cancer diagnosis—Results from a randomized controlled trial
Source: Digit Health. 2024 Dec 26;10:20552076241310037. doi: 10.1177/20552076241310037 (PMC11686631; doi:10.1177/20552076241310037)
Supplement: sj-docx-2-dhj-10.1177_20552076241310037 - Supplemental material for Efficacy of a web-based psychoeducational intervention targeting young adults with sexual problems 1.5 years after cancer diagnosis—Results from a randomized controlled trial [file sj-docx-2-dhj-10.1177_20552076241310037.docx]

**Supplementary Figures A-R:** Within group analyses on the interaction effect (level of sexual dysfunction at baseline*time). Linear mixed models with subject specific random intercept in the selected SexFS domains at post-intervention (T1) and three-month later (T2) compared to baseline (T0).


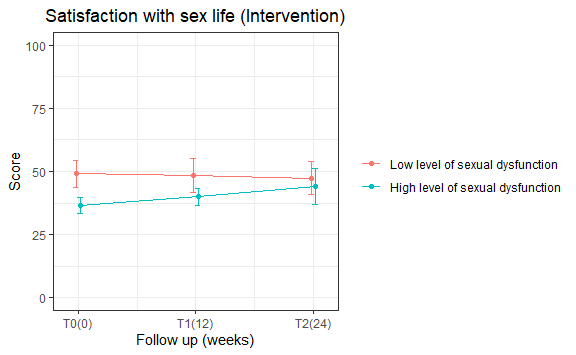


**Figure A**. Statistically significant changes compared to T0 among those with high level of sexual dysfunction (>1 SD below the mean at T0). T1 (n=20), p=0.017; T2 (n=20), p<0.001. No signifiant changes were seen among participants with low level of sexual dysfunction at baseline.


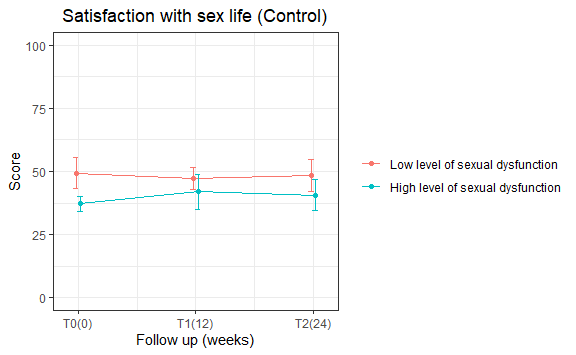


**Figure B**. Statistically significant changes compared to T0 among those with high level of sexual dysfunction (>1 SD below the mean at T0). T1 (n=29), p<0.001; T2 (n=22) p=0.020. No signifiant changes were seen among participants with low level of sexual dysfunction at baseline.


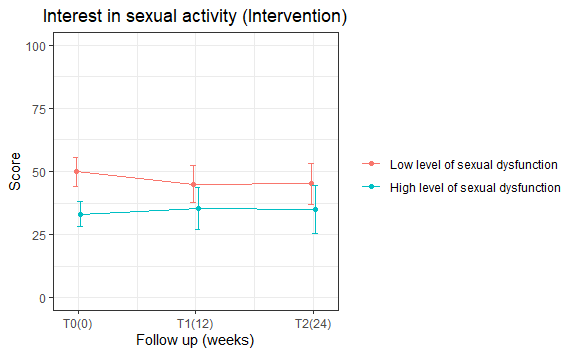


**Figure C.** Statistically significant changes compared to T0 among those with high level of sexual dysfunction (>1 SD below the mean at T0). T1 (n=31), p=0.001; T2 (n=26) p=0.004. No signifiant changes were seen among participants with low level of sexual dysfunction at baseline.


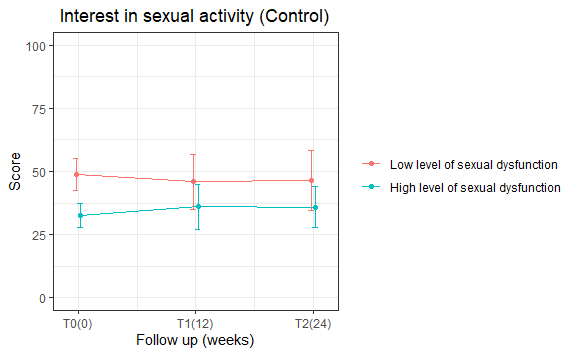


**Figure D.** Statistically significant changes compared to T0 among those with high level of sexual dysfunction (>1 SD below the mean at T0). T1 (n=34) p=0.012; T2 (n=29), p=0.037. No signifiant changes were seen among participants with low level of sexual dysfunction at baseline.


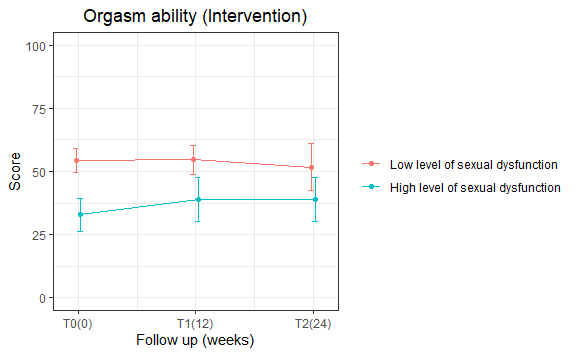


**Figure E.** Statistically significant changes compared to T0 among those with high level of sexual dysfunction (>1 SD below the mean at T0). T1 (n=16) p=0.035; T2 (n=16), p=0.001. No signifiant changes were seen among participants with low level of sexual dysfunction at baseline.


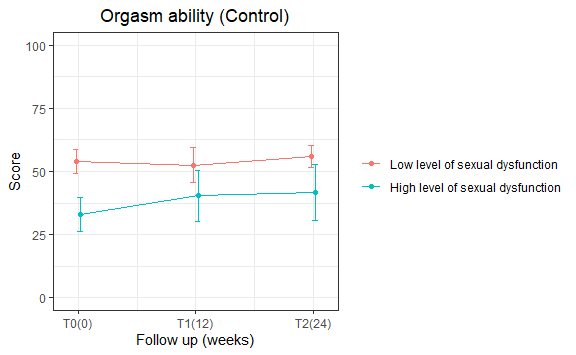


**Figure F.** Statistically significant changes compared to T0 among those with high level of sexual dysfunction (>1 SD below the mean at T0). T1 (n=25), p<0.001; T2 (n=14), p=0.010. No signifiant changes were seen among participants with low level of sexual dysfunction at baseline.


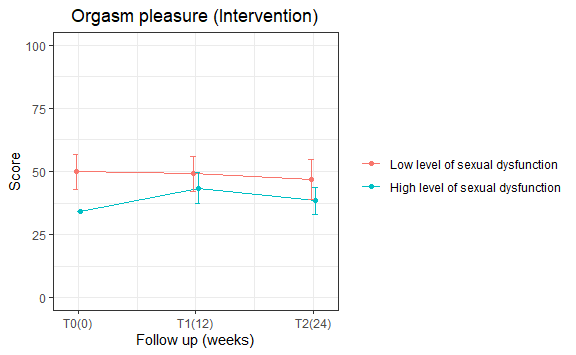


**Figure G.** Statistically significant changes compared to T0 among those with high level of sexual dysfunction (>1 SD below the mean at T0). T1 (n=5) p<0.001; T2 (n=6), p=0.024. No signifiant changes were seen among participants with low level of sexual dysfunction at baseline.


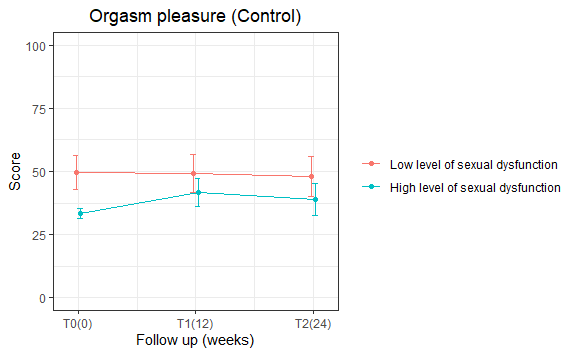


**Figure H.** Statistically significant changes compared to T0 among those with high level of sexual dysfunction (>1 SD below the mean at T0). T1 (n=8), p=0.013; T2 (n=6). No signifiant changes were seen among participants with low level of sexual dysfunction at baseline.


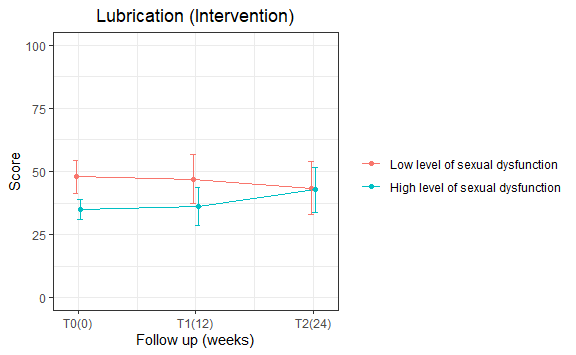


**Figure I.** Statistically significant changes compared to T0 among those with high level of sexual dysfunction (>1 SD below the mean at T0). T1 (n=22); T2 (n=16,) p<0.001. No signifiant changes were seen among participants with low level of sexual dysfunction at baseline.


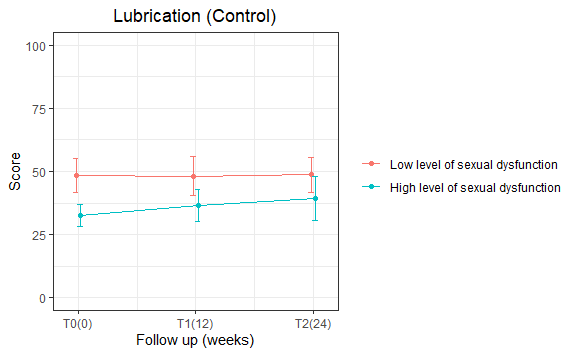


**Figure J.** Statistically significant changes compared to T0 among those with high level of sexual dysfunction (>1 SD below the mean at T0). T1 (n=20), T2 (n=15). No signifiant changes were seen among participants with low level of sexual dysfunction at baseline.


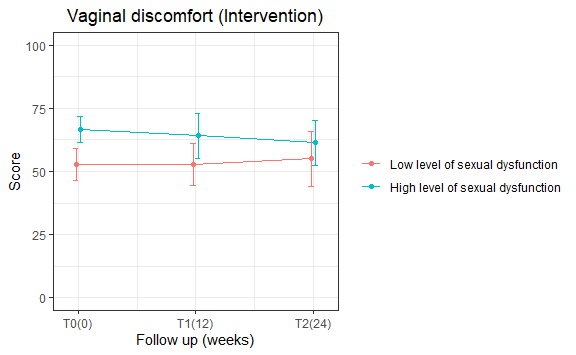


**Figure K**. Statistically significant changes compared to T0 among those with high level of sexual dysfunction (>1 SD below the mean at T0). T1 (n=13), T2 (n=12), p=0.047. No signifiant changes were seen among participants with low level of sexual dysfunction at baseline.


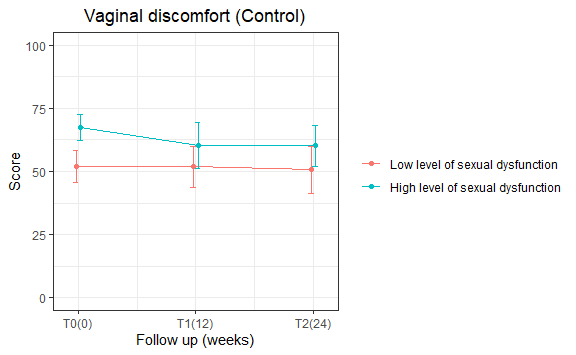


**Figure L**. Statistically significant changes compared to T0 among those with high level of sexual dysfunction (>1 SD below the mean at T0). T1 (n=16), p=0.003; T2 (n=12). No signifiant changes were seen among participants with low level of sexual dysfunction at baseline.


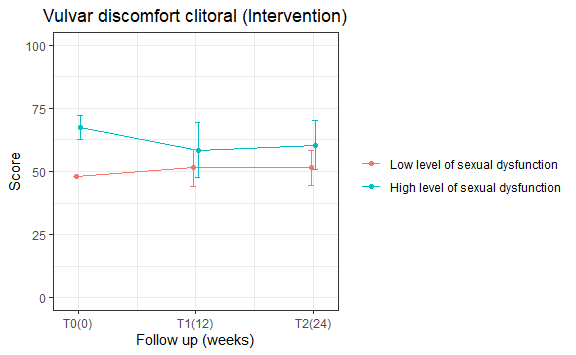


**Figure M**. Statistically significant changes compared to T0 among those with high level of sexual dysfunction (>1 SD below the mean at T0). **IG:** T1 (n=24), p<0.001; T2 (n=17), p<0.001. No signifiant changes were seen among participants with low level of sexual dysfunction at baseline.


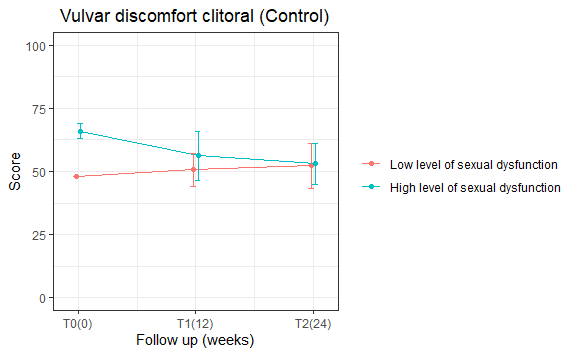


**Figure N**. Statistically significant changes compared to T0 among those with high level of sexual dysfunction (>1 SD below the mean at T0). T1 (n=14); T2 (n=7), p<0.001. No signifiant changes were seen among participants with low level of sexual dysfunction at baseline.


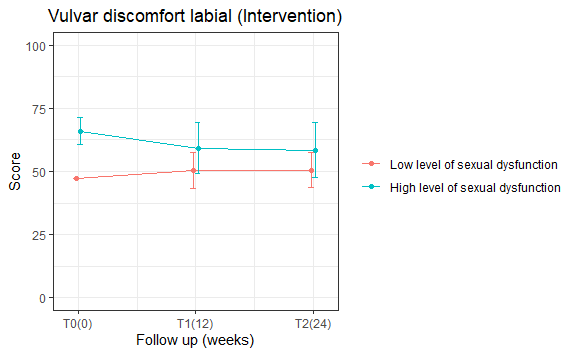


**Figure O**. Statistically significant changes compared to T0 among those with high level of sexual dysfunction (>1 SD below the mean at T0). T1 (n=27); T2 (n=16,) p<0.001. No signifiant changes were seen among participants with low level of sexual dysfunction at baseline.


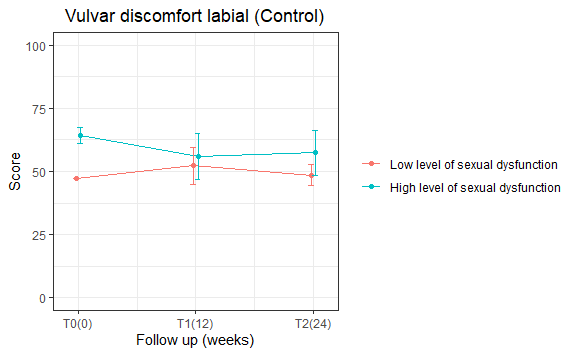


**Figure P**. Statistically significant changes compared to T0 among those with high level of sexual dysfunction (>1 SD below the mean at T0). T1 (n=24), p<0.001; T2 (n=15), p=0.004. No signifiant changes were seen among participants with low level of sexual dysfunction at baseline.


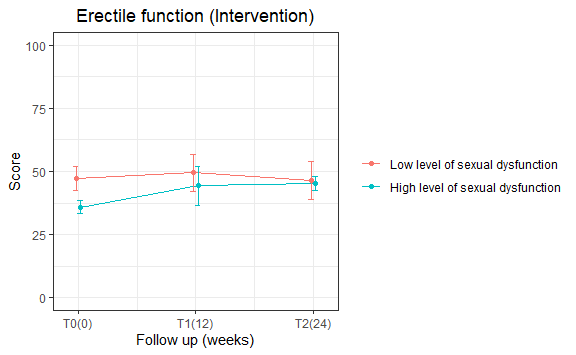


**Figure Q.** No statistically significant changes compared to T0 were seen among those with high level of sexual dysfunction (>1 SD below the mean at T0). T1 (n=4); T2 (n=4). No signifiant changes were seen among participants with low level of sexual dysfunction at baseline.


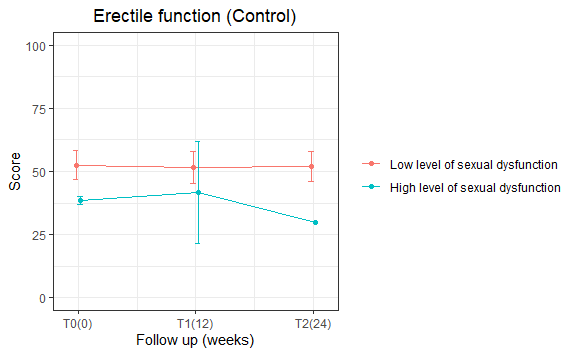


**Figure R.** No statistically significant changes compared to T0 were seen among those with high level of sexual dysfunction (>1 SD below the mean at T0). T1 (n=2); T2 (n=1). No signifiant changes were seen among participants with low level of sexual dysfunction at baseline.
